# The Impact of the Demographic Transition on Dengue in Thailand: Insights from a Statistical Analysis and Mathematical Modeling

**DOI:** 10.1371/journal.pmed.1000139

**Published:** 2009-09-01

**Authors:** Derek A. T. Cummings, Sopon Iamsirithaworn, Justin T. Lessler, Aidan McDermott, Rungnapa Prasanthong, Ananda Nisalak, Richard G. Jarman, Donald S. Burke, Robert V. Gibbons

**Affiliations:** 1 Department of Epidemiology, Johns Hopkins Bloomberg School of Public Health, Baltimore, Maryland, United States of America; 2 Bureau of Epidemiology, Ministry of Public Health, Nonthaburi, Thailand; 3 Department of Biostatistics, Johns Hopkins Bloomberg School of Public Health, Baltimore, Maryland, United States of America; 4 Armed Forces Research Institute of Medical Sciences, Bangkok, Thailand; 5 Department of Epidemiology, Graduate School of Public Health, University of Pittsburgh, Pittsburgh, Pennsylvania, United States of America; Oxford University Clinical Research Unit, Viet Nam

## Abstract

**Background:**

An increase in the average age of dengue hemorrhagic fever (DHF) cases has been reported in Thailand. The cause of this increase is not known. Possible explanations include a reduction in transmission due to declining mosquito populations, declining contact between human and mosquito, and changes in reporting. We propose that a demographic shift toward lower birth and death rates has reduced dengue transmission and lengthened the interval between large epidemics.

**Methods and Findings:**

Using data from each of the 72 provinces of Thailand, we looked for associations between force of infection (a measure of hazard, defined as the rate per capita at which susceptible individuals become infected) and demographic and climactic variables. We estimated the force of infection from the age distribution of cases from 1985 to 2005. We find that the force of infection has declined by 2% each year since a peak in the late 1970s and early 1980s. Contrary to recent findings suggesting that the incidence of DHF has increased in Thailand, we find a small but statistically significant decline in DHF incidence since 1985 in a majority of provinces. The strongest predictor of the change in force of infection and the mean force of infection is the median age of the population. Using mathematical simulations of dengue transmission we show that a reduced birth rate and a shift in the population's age structure can explain the shift in the age distribution of cases, reduction of the force of infection, and increase in the periodicity of multiannual oscillations of DHF incidence in the absence of other changes.

**Conclusions:**

Lower birth and death rates decrease the flow of susceptible individuals into the population and increase the longevity of immune individuals. The increase in the proportion of the population that is immune increases the likelihood that an infectious mosquito will feed on an immune individual, reducing the force of infection. Though the force of infection has decreased by half, we find that the critical vaccination fraction has not changed significantly, declining from an average of 85% to 80%. Clinical guidelines should consider the impact of continued increases in the age of dengue cases in Thailand. Countries in the region lagging behind Thailand in the demographic transition may experience the same increase as their population ages. The impact of demographic changes on the force of infection has been hypothesized for other diseases, but, to our knowledge, this is the first observation of this phenomenon.

*Please see later in the article for the Editors' Summary*

## Abstract 2

Analyzing data from Thailand's 72 provinces, Derek Cummings and colleagues find that decreases in birth and death rates can explain the shift in age distribution of dengue hemorrhagic fever.

## Editors' Summary

**Background:**

Every year, dengue infects 50–100 million people living in tropical and subtropical areas. The four closely related viruses that cause dengue are transmitted to people through the bites of female *Aedes aegypti* mosquitoes, which acquire dengue virus by feeding on the blood of an infected person. Although some people who become infected with dengue virus have no symptoms, many develop dengue fever, a severe, flu-like illness that lasts for a few days. Other people—more than half a million a year—develop dengue hemorrhagic fever, which causes bleeding from the gums and nose and bruising, or dengue shock syndrome in which circulatory failure also occurs. Both these potentially fatal conditions are associated with sequential infections with dengue virus—nonfatal infection with dengue virus of one type provides lifelong immunity against that type but only temporary protection against infection with dengue viruses of other types. There is no vaccine to prevent dengue and no specific treatment for the disease. However, standard medical care—in particular, replacement of lost fluids—can prevent most deaths from dengue.

**Why Was This Study Done?:**

Historically, dengue has mainly affected young children but, recently, its age distribution has shifted towards older age groups in several Southeast Asian countries, including Thailand. In addition, the interval between large increases in incidence (epidemics) of dengue hemorrhagic fever has lengthened. It is important to know why these changes are happening because they could affect how dengue infections are dealt with in these countries. One idea is that an ongoing shift towards lower birth and death rates (the demographic transition; this occurs as countries move from a pre-industrial to an industrial economy) is reducing dengue transmission rates by reducing the “force of infection” (the rate at which susceptible individuals become infected). As birth and death rates decline, immune individuals account for more of the population so mosquitoes are more likely to bite an immune individual, which reduces the force of infection. Similarly, because susceptible individuals enter the population by being born, changing the birth rate alters the interval between epidemics. In this study, the researchers test whether the demographic transition might be responsible for the changing pattern of dengue infection in Thailand.

**What Did the Researchers Do and Find?:**

The researchers retrieved data on dengue infection, demographic data (the population's age structure and birth and death rates), socioeconomic data, and climatic data for Thailand from 1980 to 2005 from various sources. They then fitted the data on dengue cases to several mathematical models to estimate the force of infection for each year. This analysis suggested that the force of infection has declined by 2% every year since the early1980s. Next, the researchers used statistical methods to show that the strongest predictor of this decline is the increase in the median age of the population (a measure of the average age of the population). Finally, using mathematical simulations of dengue transmission, they showed that a reduced birth rate and a shift in the population's age structure are sufficient to explain the recent shift in the age distribution of dengue cases, the reduction of the force of infection, and the increased interval between epidemics of dengue hemorrhagic fever.

**What Do These Findings Mean?:**

The findings of all modeling studies depend on how the mathematical models are built and the accuracy of the data fed into them. Nevertheless, these findings suggest that recent changes in birth and death rates in Thailand are sufficient to produce the observed changes in the age distribution of dengue and periodicity of dengue outbreaks. One implication of these findings is that other countries in Southeast Asia that follow Thailand in the demographic transition may experience similar shifts in the pattern of dengue infections as the age structure of their populations changes. This means that clinical guidelines for the management of dengue infections in Southeast Asia will need to be adjusted to allow for the increasing age of dengue cases. Finally, although the researchers' calculations show the force of infection has fallen substantially over the past two decades, they also show that when a dengue vaccine becomes available, it will still be necessary to vaccinate most of the population to halt dengue transmission.

**Additional Information:**

Please access these Web sites via the online version of this summary at http://dx.doi.org/10.1371/journal.pmed.1000139

- This study is further discussed in a *PLoS Medicine* Perspective by Cameron Simmons and Jeremy Farrar
- The US Centers for Disease Control and Prevention provides detailed information about dengue fever and dengue hemorrhagic fever (in English and Spanish)
- The World Health Organization provides information about dengue and dengue hemorrhagic fever around the world (in several languages) and detailed information about dengue in Southeast Asia
- Links to additional information about dengue are provided by MedlinePlus (in English and Spanish)
- Wikipedia has a page about the demographic transition (note that Wikipedia is a free online encyclopedia that anyone can edit; available in several languages)

## Introduction

In Thailand, dengue fever (DF) and dengue hemorrhagic fever (DHF) have traditionally been diseases of childhood, causing substantial morbidity and mortality among children <15 y of age. Recently, an increasing number of DHF cases among older individuals has been observed in Thailand [1],[2]. In several Southeast Asian countries the age distribution of dengue cases detected by passive surveillance has shifted towards older age groups [3]–[5]. This shift has implications for clinical diagnosis and management, and may present new challenges to dengue control. Identifying the mechanism responsible for this age shift would enable us to forecast whether this trend is likely to continue, understand its impact on public health, and identify policies that may reduce morbidity and mortality from dengue.

There are several potential reasons for this shift. One hypothesis is that the force of infection (the per capita rate at which susceptible individuals become infected) is declining because of reductions in vector abundance or contact between human and vector, possibly as a result of control efforts or economic development [2],[6]. A reduced force of infection would increase the average age of infection, a well-known result for many other diseases [7].

Another hypothesis is that the age distribution of dengue cases has not changed at all, but reporting has shifted over time. The national reporting system for dengue receives reports of DHF, dengue shock syndrome (DSS) and, since 1994, DF. There has been a shift in the Thai surveillance system to report a larger number of cases of DF. Differences in the age distribution of this newly reported class of clinical cases could increase the apparent average age of dengue cases.

**Figure 1:**
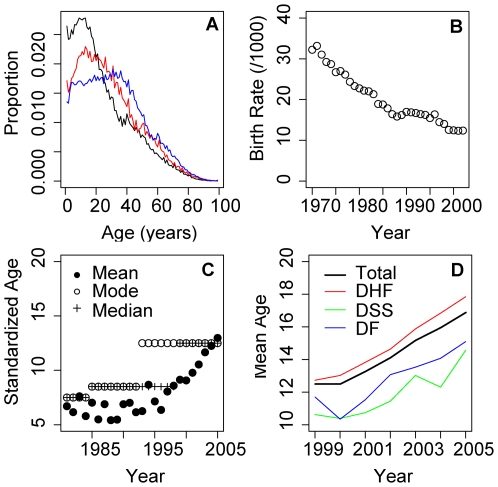
Demographic changes in Thailand and changes in the age distribution of dengue illness. (A) Age structure of the Thai population 1980 (black), 1990 (red), and 2000 (blue). (B) Birth rates per 1,000 individuals 1970–2000. (C) Mean, median, and modal age of age-standardized dengue incidence data in Thailand 1981–2005. Age-specific incidence in all years was standardized to age distribution of Thailand's population in 2000 using direct age adjustment. Mean (filled circles), mode (unfilled circles), and median (plus sign) age of standardized age-specific incidence of dengue disease reported to the national surveillance system for each year are shown. (D) Mean age of DHF (red), DSS (green), DF cases (blue), and total (black) cases reported to the national surveillance system 1999–2005.

Here, we hypothesize that the shift in the age distribution of dengue cases is due to a shift in the age structure of the population, and its impact on the force of infection. Over the last 20 y the age structure of Thailand has changed dramatically. In 1980, 40% of the population was under 15 y; in 2000, 24% were under 15 y (Figure 1A) [8],[9]. Birth rates have declined from 32/1,000 in 1970 to 12/1,000 in 2005 (Figure 1B) [8],[10]. Changes in the age structure may significantly alter transmission [11]. As birth and death rates decline, immune individuals become a larger proportion of the population. Hence, mosquitoes are proportionally more likely to bite immune individuals, and the force of infection is reduced. The increased proportion of immune individuals protects susceptible individuals and delays their infection until a later date.

The age distribution of reported dengue cases provides information on the level of transmission occurring in the population. The age at which individuals become infected can be used to estimate the hazard of infection and the age-specific risk of infection [12],[13].

In this paper, we investigate changes in the age distribution of dengue disease reported to the Thai national surveillance system from 1985 to 2005. We test whether the change in the age distribution of dengue cases is attributable to changes in the distribution of dengue disease among the three clinical presentations (DHF, DF, DSS) of dengue infection or due to a change in the age distribution of just one of these clinical presentations.

We estimate the force of infection of dengue in each of the provinces of Thailand and look for associations between the spatial and temporal distribution of the force of infection and socio-demographic characteristics including age structure, birth rate, climate factors, and economic indices. An association between the force of infection and demographic structure supports our hypothesis that the demographic transition is the cause of the observed increase in the average age of dengue cases.

Changes in birth rate have been shown to be an important driver of changes in the dynamics of childhood infectious diseases [14]. DHF incidence in Thailand exhibits a strong 2–4-y periodicity [15]. Several authors have hypothesized that this oscillation is due to predator–prey dynamics between the pathogen and the host population [16],[17]. Birth is the primary way susceptible individuals enter the population. Changing birth rates are expected to change the period of oscillations even if the rate at which infectious contacts are made and the probability that those contacts result in transmission stay the same. We investigate changes in the period of oscillations in DHF incidence and its relation to changes in socioeconomic, demographic, and climatic indices using statistical analysis and theoretical models of dengue transmission.

## Methods and Approach

### Data

A reporting system for communicable disease that includes dengue was established in Thailand by the Ministry of Public Health in the early 1970s [18]. Dengue cases identified by clinicians working in central, general, community, and some private hospitals are reported to provincial health officers, aggregated at the provincial level, and reported to the Bureau of Epidemiology, Department of Disease Control, Ministry of Public Health. Before 1994 only DHF and DSS cases were reported, and from 1994 on DF, DHF, and DSS were reported. Before 1984 cases were aggregated into ten age classes, and into 16 age classes from 1985 on. Our analysis uses data from 1980 to 2005. Data from this entire time period were available for 72 of the 76 provinces of Thailand.

Population data for each province of Thailand were obtained from censuses in 1980, 1990, and 2000 performed by the National Statistical Office of Thailand [8],[9],[19]. Populations for noncensus years were estimated by fitting a log-linear model to census years. We obtained climate data from the National Oceanic and Atmospheric Administration's Climate Anomaly Monitoring System [20]. We obtained socioeconomic data from the 1980, 1985, 1990, and 2000 Census of Thailand as well as the Statistical Yearbook of Thailand in multiple years [9],[19].

### Estimating the Force of Infection

We characterize the level of transmission in each province using the force of infection at time *t*, λ(*t*). The force of infection has been found to vary by age and time for several pathogens [21]. Using serological survey or age-stratified case data from one time point one can estimate either the force of infection of serotype *i* in age group *a*, λ*_i_*(*a*), or the force of serotype *i* in time period *t*, λ*_i_*(*t*). Using available case data from multiple years, we estimate the force of infection indexed by both age and time, λ*_i_*(*a*,*t*) [22]. Thai national surveillance data do not specify the serotype of infections, so only the sum and mean of λ*_i_*(*a*,*t*) across all serotypes can be estimated. In the rest of this manuscript we use 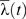 to refer to the mean force of infection at time *t* and 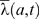 if specific to an age and time.

Using age-stratified case reports, as opposed to serological data, to estimate 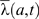 assumes that the age distribution of reported disease provides an unbiased estimate of the age distribution of infections. We assume case reports provide an unbiased estimate of the age distribution of secondary infections, rather than primary infections, since most clinically apparent dengue illnesses are secondary infections [23]. Several studies in Thailand have found that secondary infections comprise approximately 90% of clinically apparent cases of dengue (100%, 88%, 97%, respectively, in the three studies) [23]–[25]. We assume that individuals infected by two different serotypes of dengue develop immunity to all other types [26]. We use the full age distribution of cases, rather than the mean age as used in other studies [6],[27], to determine whether the shift in the age of dengue cases reflects a shift in incidence in a subset of age groups or in all age groups. This approach allows us to estimate differences in the risk of infection by age and by immune status.

We fit the age distribution of dengue cases in each province in each year using multiple models. We assumed that the cumulative proportion of age-specific incidence per individual occurring up until each age provides an estimate of the proportion of individuals who have experienced two infections as a function of age. We then used catalytic models that have been applied to age-specific serological data to estimate the force of infection in each year for which we have age-specific dengue incidence (1985–2005), as well as the 20 y preceding [22]. We used multiple approaches, including models that estimate age-specific forces of infection and increases in the susceptibility of those who have experienced a primary infection. We denoted the models we used model 1–4. Model 1 assumes that the force of infection was constant over time. This is the simplest model we estimated and was used to assess the fit of other, more detailed models. Model 2 includes 41 free parameters, one λ(*t*) for each year of the dataset (1985–2005) and for each year that the oldest individuals (20) in the first year of data were alive and at risk of acquiring dengue (1965–1984). Model 3 includes 51 free parameters, the time-dependent forces of infection of model 2 and an additional ten age-specific additive factors that modify the risk of individuals by age. Model 4 includes 51 free parameters, the time-dependent forces of infection of model 2 and an additional ten age-specific multiplicative factors that modify the risk of individuals by age. Several additional alternatives were considered in sensitivity analysis. Details on each of these models are included in Text S1.

We used inverse variance–weighted linear regression to estimate the association of the mean force of infection in each province (1985–2005) and socioeconomic, demographic, and climatic factors. Similar methods were used to associate the change in force of infection in each province from 1985 to 2005 with changes in socioeconomic, demographic, and climatic factors.

We used wavelet analysis to estimate the multiannual periodicity of incidence in each of the provinces of Thailand. Wavelet transforms give an estimate of the period of oscillations at each point in a time series [28]. We used inverse variance–weighted linear regression to estimate the association between the period of oscillations and the same list of covariates used in the regression of force of infection above, considering associations with both mean period and changes in period between 1985 and 2005.

### Simulation Models

We developed deterministic models of the transmission dynamics of four dengue serotypes to examine the impact of reducing birth rates on the period of oscillations in incidence. We considered three models: one with immune enhancement of transmission [16], one with short-term cross protection from heterotypic infection [29], and one with seasonal variation in transmissibility (see Text S1, Simulations). Because we hypothesize that changes in the human, rather than the mosquito population account for changes in dengue dynamics, the vector population was not modeled explicitly.

We developed an age-specific model of dengue transmission to explore the impact of secular demographic trends (i.e., changing birth and death rates) on transmission dynamics and the age distribution of cases [11],[30]. This is a two-serotype model with ten age classes and independent birth and death rates. We restricted the model to two serotypes for simplicity, but expect a four-serotype model would show similar results.

## Results

Standardized age-specific incidence rates (see Text S1, Age Standardization) show a substantial increase in the average age of dengue cases (Figure 1C). Increases in average age have been observed for DHF, DF, and DSS (Figure 1D), with DHF cases having a slightly higher average age than DF and DSS cases.

**Figure 2:**
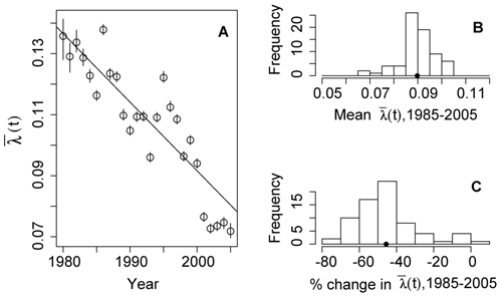
The force of infection of dengue in Thailand, 1980–2005. (A) Estimates of the force of infection (per year) for each year, 1980–2005 in Thailand estimated using age-specific case data aggregated at the national level and model 3 (see Text S1). 95% CIs are indicated by vertical bars. The line drawn is the least squares linear regression weighted using the reciprocal of width of CIs on each point. This linear trend shows a reduction in the force of infection of 0.002 per year (*p*<1e−6). (B) Histogram of mean forces of infection averaged over the interval 1985–2005 estimated using data from each of the 72 provinces of Thailand. Histogram shows the frequency of 72 estimates occurring in the ranges indicated on the *x*-axis. (C) Histogram of changes in forces of infection over the interval 1985–2005 estimated using data from each of the 72 provinces of Thailand. Histogram shows the frequency of 72 estimates of the change in force of infection occurring in the ranges indicated on the *x*-axis. We estimated changes using weighted least squares regression of annual estimates of the force of infection in each location.

Figure 2A shows estimates of 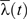 for 1985–2005. 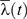 has declined roughly linearly over this interval with a reduction from 0.14 to 0.07. This trend is statistically significant with a reduction of −2.4e−3 in the force of infection per year (95% confidence interval [CI] −1.9e−3 to −2.9e−3). The mean force of infection over the interval 1985–2005 is 0.10, thus each serotype infects 10% of susceptible individuals each year. The mean force of infection from 1985 to 2005 varies across provinces, ranging from 0.07 to 0.10 with a mean of 0.09 (Figure 2B), and has declined in every province of Thailand with a mean decline of 45.6% (range 70% decrease, 6% increase) (Figure 2C).

**Figure 3:**
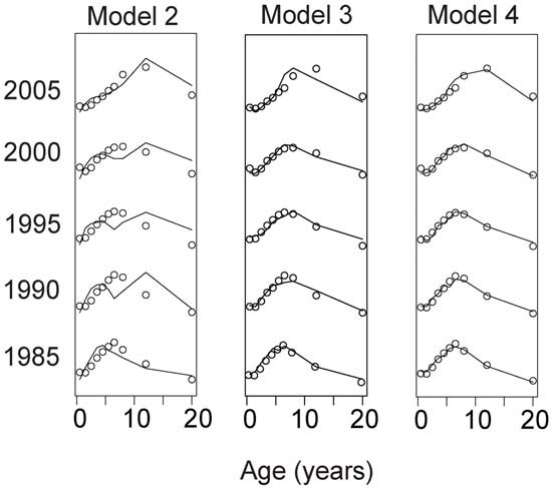
Fit of models to data 1985, 1990, 1995, 2000, 2005, model 2, 3, and 4. Age-specific incidence is shown by circles and fits from model shown by lines. Model 2 includes time-specific but not age-specific forces of infection. Model 3 includes additive age-specific forces of infection in addition to time-specific forces. Model 4 includes multiplicative age-specific forces of infection in addition to time-specific forces.

The best fitting model of the four estimated was model 4, which includes multiplicative age factors that modify the force of infection. The estimates of the force of infection generated by this model appear in Figure 2A–2C. Compared to a model with a constant force of infection in each province, the inclusion of time-specific forces of infection improved the fit of the model significantly (chi-square test of difference in likelihoods, *p*<0.001). Text S1, table S1 summarizes each of the models estimated and measures of their goodness of fit. The fit of three of these models to the age-specific incidence data for 5 y is found in Figure 3. Visually, the models fit the data well. The figure shows that models that include age-specific factors (models 3 and 4) fit much better than model 1, which does not. Age-specific relative hazards compared to baseline reach peaks in the 0–1-y-old groups (2.12 95% CI 2.04–2.21) and 7–9-y-old age groups (1.94 95% CI 1.89–1.98) (Text S1, figure S1). Children 2–3 y old have a low relative hazard of infection (0.81 95% CI 0.78–0.84), as do older individuals, with individuals 14–20 y old having a multiplicative factor of 0.05 (95% CI 0.02–0.24). Though the fit of these models varies significantly, all yielded consistent estimates of the secular trends in the force of infection for the country-wide data and for each province. Plots of estimates generated with alternative models shown in Text S1 show that the models produce very similar estimates of the force of infection over time.

In Rayong province both age-specific DHF incidence and age-stratified serological data for a single year (1980) are available [20]. Using these data, our estimates of the force of infection averaged over 1969–1979 are slightly higher than estimates from serological data appearing in [22] (0.12 versus 0.09), but within the 95% CI (see Text S1).

Time-specific estimates of the force of infection exhibit multiannual oscillations as characterized by Fourier spectra. 48 of 72 provinces had significant peaks in Fourier spectra of time series of estimates of the force of infection generated by model 4 between a period of 2.5 and 6 y. The most common peak in Fourier spectra in this range was 2.7 y. This estimate is consistent with mean multiannual periodicities of DHF incidence data in Thailand [15].

Including increased susceptibility of previously exposed individuals significantly improved the model fit to country-wide data (chi-square test of difference in likelihoods, each *p*<0.001) but did not improve the fit considering all provinces. Text S1, figure S3 shows the fit of this model to the country-wide aggregate data in four time points.

### 

#### Association of the force of infection with socio-demographic and climatic factors

Our central hypothesis is that increases in the median age of the total population lead to decreases in the force of infection. Province-specific mean forces of infection from 1985 to 2005 generated using model 4 were significantly associated with the provincial median age, with an estimated decrease of 1.5×10^−3^ (95% CI 8.7×10^−4^, 2.2×10^−3^) per year increase in the median age of provincial population. Mean provincial force of infection was significantly associated with mean household size, birth rate, proportion of the population under 15 y of age, percent of homes with sanitation, latitude, and average monthly rainfall (Table 1). Median age and mean force of infection remain significantly associated in a multivariate model including all other statistically significant covariates.

**Table 1:** Results of univariate linear regression of force of infection estimated using model 4 on demographic, socioeconomic, and climatic characteristics of provinces.

Independent Variable	Regression coefficients (95% CI)
Median age	−1.5e−3 (−2.2e−3 to −8.7e−4)
Mean household size	9.7e−3 (4.8e−3 to 1.5e−2)
Birth rate (per 1,000)	4.2e−4 (8.3e−6 to 8.3e−4)
Proportion of population under 15 y of age	6.8e−2 (3.7e−2 to 1.0e−1)
Percent of homes with sanitation	−2.1e−4 (-3.3e−4 to −8.3e−5)
Average rainfall (mm/mo)	4.2e−5 (3.1e−6 to 8.1e−5)
Latitude (degrees)	−1.3e−3 (-1.6e−3 to −8.1e−4)

School attendance, percent of homes constructed with permanent materials, percent of provincial population in urban areas, gross provincial product (Baht per capita), and average temperature were not statistically significantly associated with the mean force of infection. Covariates significant in multiple linear regression: median age −3.2e−3 (95% CI −5.9e−3 to −5.1e−4), latitude −1.1e−8 (95% CI −2.0e−8 to −1.4e−9), and birth rate −1.4e−3 (95% CI −2.3e−3 to −4.1e−4).

The change in force of infection was significantly associated with the changes in two covariates, median age and the percentage of homes constructed with permanent materials (see Table 2). In univariate analysis, each year increase in the median age of the population was associated with a −4.6e−2 change in 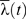 (95% CI −7.7e−2 to −1.5e−2) and a percentage increase in homes built with permanent materials was associated with a −1.5e−2 change in 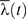 (95% CI −2.6e−2 to −3.6e−3).

**Table 2:** Results of univariate linear regression of change in the force of infection from 1985 to 2005 estimated using model 4 on demographic, socioeconomic, and climatic characteristics of provinces.

Independent Variable	Regression Coefficients (95% CI)
Change in median age	−4.6e−2 (−7.7e−2, −1.5e−2)
Change in percentage of homes constructed with permanent materials	−1.5e−2 (−2.6e−2, −3.6e−3)

Change in school attendance, household size, percent of provincial population in urban areas, birth rate, gross provincial product, average rainfall, and average temperature were not statistically significantly associated with the change in the mean force of infection. Both median age and change in percentage of homes constructed with permanent materials significant in model that includes both.

Only median age was found to be a statistically significant predictor of both the mean force of infection and the change in the force of infection in each province between 1985 and 2005.

### Changes in Incidence

**Figure 4:**
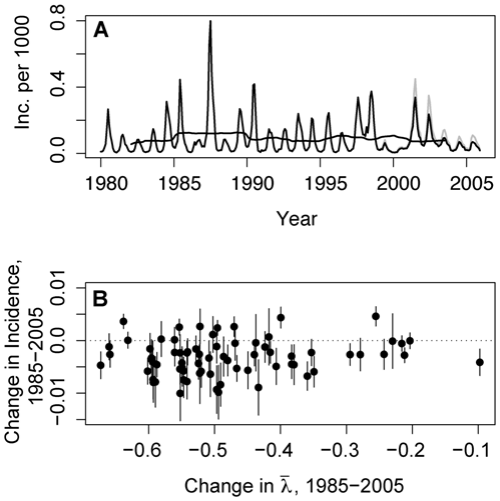
Incidence of dengue disease in Thailand, 1981–2005. (A) Incidence of dengue disease in Thailand, 1981–2005. The solid line shows the incidence per 1,000 individuals per year of DHF and DSS together, whereas the grey line shows the incidence of DHF, DSS, and DF. DF was included in case reports starting in 1993. (B) Change in incidence (per 1,000 per year) 1985–2005 with 95% CI versus change in force of infection for each of the provinces of Thailand. A linear association between these two variables is not statistically significant.

Between 1985 and 2005, there was a small but statistically significant decline in the national incidence of DHF (Figure 4A). The majority of individual provinces have also shown significant declines, but these declines are not significantly associated with changes in force of infection (Figure 4B). Considering a broader interval (1980–2005), the reduction in annual incidence is not significant.

### Changes in Periodicity

**Figure 5:**
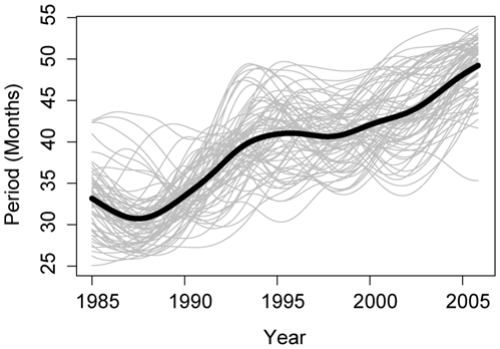
Period of multiannual oscillations of DHF incidence in each of the 72 provinces of Thailand. Each line is the period in months of the incidence in one province. Period presented is the mean period of power in a period band of 18 to 60 mo reconstructed using the continuous wavelet transform (see Text S1, Detailed Methods). The thick line shows the period of multiannual oscillations of country-wide incidence.

Figure 5 shows the period of multiannual oscillations (reconstructed from a continuous wavelet transform using a period band of 1.5 y and 5 y) as a function of time for each province of Thailand. As noted elsewhere [7], most provinces have experienced a lengthening in the period of oscillations. The oscillations appear to undergo meta-oscillations in period (the multiannual period oscillates from 2–4 y every 10 y). We examined the relationship between a linear trend in period estimated by wavelet analysis and the socioeconomic, demographic, and climatic variables listed in Table 1. Two covariates are significantly associated with changes in periodicity: there is a 8.6e−2-month (95% CI 2.2e−2 to 1.5e−1) increase in period with each year increase in provincial median age and a 1.6e−2-month (95% CI 2.0e−3 to 3.0e−2) increase in period with each percentage increase in school attendance among school-aged children from 1985 to 2005. Results are shown in Table 3. Changes in school attendance are highly correlated with the changes in median age. A linear model including both covariates shows a statistically significant association between median age and periodicity, but not between school attendance and periodicity.

**Table 3:** Results of univariate linear regression of change in the period of multiannual oscillations from 1985 to 2005 on demographic, socioeconomic, and climatic characteristics of provinces.

Independent Variable	Regression Coefficients (95% CI)
Change in median age	0.09 (0.02–0.15)
Change in school attendance	0.02 (0.002–0.03)

Change in mean household size, percent of provincial population in urban areas, birth rate, percentage of homes constructed with permanent materials, gross provincial product, average rainfall, and average temperature were not statistically significantly associated with the change in period of multiannual oscillations.

### The Basic Reproductive Number and the Critical Vaccination Fraction

Using the forces of infection, we estimated the basic reproductive number (*R*_0_) of dengue in each province. *R*_0_ characterizes the intrinsic transmissibility of the pathogen in a setting, as opposed to the force of infection, which depends upon the number of individuals currently infectious [8]. A primary use of *R*_0_ is to estimate the fraction of the population that must be vaccinated to eliminate transmission, the critical vaccination fraction, *v* (*v* = *1*−*1/R*_0_). We estimate the decline in the mean critical vaccination fraction in Thailand from 1980 to 2005 to be 5%, from 85% to 80% (i.e., *R*_0_ has declined from 6.7 to 5.2). The critical vaccination fraction depends on the fraction susceptible at equilibrium. We estimate that the fraction of the population that is susceptible to any dengue serotype has changed little over the last 25 y, increasing from 14% to 19% of the population.

### Transmission Models

**Figure 6:**
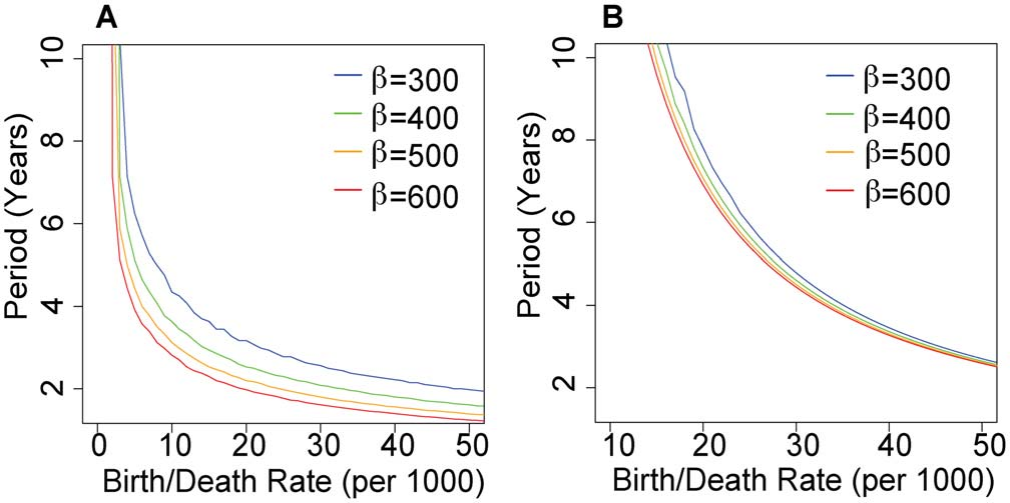
Period of multiannual oscillations of a four-serotype dengue transmission model. (A) Simulations include immune enhancement of transmission (second infections are 1.85 times as transmissible as primary infections) [17]. (B) Simulations including no immune enhancement but including seasonality in transmission coefficient (peak coefficients exceed mean coefficients by 4%). Period is plotted as a function of birth/death rate for simulations in which the transmission coefficient, β is 300 (blue), 400 (green), 500 (orange), and 600 (red).

The period of multiannual oscillations in simulations of dengue transmission are dependent on assumed birth and death rates. Figure 6A shows the period of multiannual oscillations as a function of birth and death rate (assumed equal, for model simplicity, but also changed independently, discussed below) for varying transmission coefficients (β), in a four-serotype model with antibody-dependent enhancement. The transmission coefficient defines the rate at which infectious contacts are made, here mediated by contact with the vector. The basic reproductive number for each strain is *R* = β/(μ+σ), where μ is the mortality rate and σ is the rate of recovery of infectious individuals. Figure 6B shows results for a model with seasonality in transmission but no antibody-dependent enhancement. As birth and death rates decline, the period separating times of high dengue incidence increases. The same qualitative behavior is seen in models that include immune enhancement and models that include short-term cross-immunity. We performed simulations using βs corresponding to a *R*_0_ of 4 to 12. At an *R*_0_ of 6 (i.e., our estimate for Thailand) a change in the birth rate similar to that observed in Thailand increases the period of the system from just over 2 y to close to 4 y. This is consistent with the observed change in the period of multiannual oscillations in dengue and DHF from a mean of 33 mo to 49 mo (Figure 5). An increase in the period of oscillations is also observed when the birth or death rate is reduced independently.

**Figure 7:**
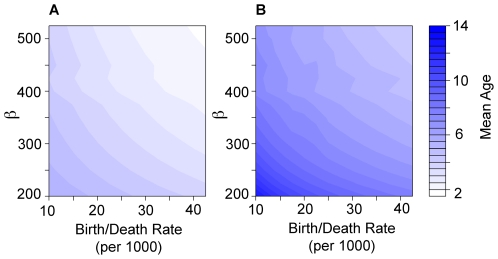
Results of an age-specific deterministic transmission model. Average age of (A) primary dengue and (B) secondary dengue cases in a two-serotype transmission model. In both plots, age is indicated by the color blue with legend at right. The birth and death rate varies from 10 per 1,000 to 40 per 1,000 and the transmission coefficient varies from 200 to 500 (*R*_0_≈4–10). The average ages of both primary and secondary cases rise with decreasing birth/death rate and decreasing transmission coefficient. The average age of secondary cases rises faster with each unit decrease in birth/death rates (range of average ages in (B), 4–16 y, range of average ages in (A) 2–5 y.

Simulations using a two-serotype model with ten age classes showed a steady increase in the average age of primary and secondary dengue infections when birth and death rates were reduced (Figure 7A and 7B). Reductions in β also increase the average age of both primary and secondary infections. There is a larger increase in the average age of secondary cases compared to primary cases as the birth/death rate or β declines (Figure 7B). As observed in the non-age-specific simulations, these models exhibit strong multiannual oscillations that increase in period with declines in β or birth/death rates.

Analysis of case data showed only a slight decrease in absolute incidence. Both the age-specific and non-age-specific simulations show large decreases in incidence as birth and death rates are reduced. However, these results are dependent upon including age-independent mortality, which is commonly used in transmission models. If instead, age-dependent mortality that is more consistent with observed age-specific rates of mortality (in the form of higher mortality among older individuals) is used, the reductions in incidence are smaller. Text S1, figure S5 shows the incidence of secondary infections in a model that includes age-independent mortality (black line) and age-dependent mortality (blue line).

## Discussion

Recent changes in the average age of DHF in Southeast Asia have been attributed to control measures [6], development [31], and changes in the distribution of serotypes or genotypes [5]. Here we present evidence of another mechanism, a change in the demographic structure of the population. We find that, in Thailand, median age is consistently associated with the (1) estimated change in the force of infection (1985–2005), (2), mean forces of infection (1985–2005) and (3) changes in the multiannual periodicity of DHF incidence. Our simulations indicate that changes in birth and death rates similar to those experienced in Thailand create changes in the age distribution of cases and the periodicity of incidence consistent with observations. Changing age structure has been suggested by other authors as a driver of reductions in childhood infectious disease mortality due to other infectious diseases in western countries in the 19th and 20th centuries [32], and as driving changes of the average age of measles infection [12]. Mechanistically, reduction in all cause mortality increases the longevity of immune individuals. These immune individuals decrease the risk of dengue infection of susceptible individuals around them by providing alternative feeding sources for infectious mosquitoes. Reduced birth rates decrease the relative abundance of susceptible individuals to immune individuals, thus further reducing the risk of infection. Serological testing could determine whether patterns of age-specific immunity are consistent with our findings using age-specific incidence data. Studies with greater geographic resolution could elucidate more specific mechanisms for changes in the force of infection. Many demographic changes accompany shifts in birth rates, death rates, and age structure, and analysis at the individual or household level is needed to understand the effect of each component of demographic change. However, our simulations indicate that only changes in birth and death rates are necessary to explain the observed changes in dengue dynamics in Thailand.

If the demographic transition is responsible for the reduction in the force of infection of dengue, a similar reduction might be seen in other diseases. The average age of chickenpox, a directly transmitted respiratory infection, has increased in Thailand over the same period, from 10.2 y in 1985 to 12.5 y in 2005 (estimated using data from the national surveillance system). The impact of demographic changes may be more easily observed in dengue than chickenpox because severe manifestations of dengue are associated with secondary exposure. The average age of secondary exposure would increase more quickly than the average age of a primary exposure with reductions in hazard because the reduced hazard delays the primary and secondary infection. Chickenpox transmission is also mediated by social interactions more strongly than dengue, as there is no vector. Thus, interactions between children sufficient to transmit chickenpox may be less affected by increases in the overall prevalence of immunity if the majority of those immune are older. The transmission models that we have used assume that contact processes are frequency dependent rather than density dependent. Density-dependent models do not show the same reductions in the force of infection because the density-dependent contact assumes that the number of immune individuals has no impact on the contact between susceptible and infectious individuals [33].

We find no evidence for the increases in annual DHF incidence between 1985 and 2000 reported in [6]. However, we do not observe the large declines in incidence that might intuitively be expected with a halving of the force of infection. Some possible explanations are changes in the efficiency of the surveillance system, higher rates of symptomatic disease among older individuals, or the impact of long-term cycles in incidence. A better explanation may be that the hazard of infection is still sufficiently large that very few individuals will remain susceptible into older adulthood. Though each individual experiences reduced risk of infection per unit of person time, each individual contributes more time at risk and thus total incidence per unit of calendar time may remain constant because of elevated incidence in older individuals. Deaths among adolescents and young adults reduce the person time that individuals contribute, but age-specific mortality rates among ten to 30 year olds are negligible and thus would not reduce case numbers substantially. Our age-specific simulations that include age-dependent mortality show only slight reductions in incidence with reductions in the birth and death rates.

The shift in the average age of infection might have public health impacts not reflected in total incidence. Total deaths due to DHF have declined steadily over the last 20 y in Thailand [34]. Although improved clinical management might contribute to this decline, increased age of infection can also reduce the case fatality rate, as the risk of mortality due to DHF declines with age [35].

There are several limitations to our study. Data from the passive surveillance describe predominantly severe, hospitalized cases. We do not have full knowledge of the biases in reporting that may be present. Ideally, our study would be based on incidence of infection as evidence by serology rather than incidence of disease. Age-stratified serological surveys performed in several locations in Thailand could help determine if the conclusions presented here are the same when considering infections rather than cases of severe disease. Another limitation of our study is that we do not have good information on reporting practices over time. To the extent that the data allow, we have found no secular trends in the types of facilities reporting or the ratio of urban to rural facilities reporting. However, we have limited ability to detect changes in reporting that could affect our results. In addition, we do not know the extent to which primary or tertiary infections may be present among the observed cases. Though they are thought to be rare among symptomatic cases, their presence in substantial numbers would bias our results.

We have not addressed a number of alternative hypotheses that could explain the observed increase in age of dengue cases. Control measures could be reducing vector densities or contact between vectors and humans. Migration may be moving adults from areas of low transmission to areas of greater transmission, thus increasing the average age of cases. Though we have identified a mechanism that can of itself explain the increase in average age and the lengthening of the interepidemic interval, we do not know what role these other factors might play in increasing the average age of dengue cases.

Several dengue vaccine candidates are under development. We estimate that both the proportion of the population susceptible and the critical vaccination fraction have changed very little (∼5%) over the last 20 y, although the force of infection has fallen substantially.

If the demographic transition is responsible for the reduction in the force of infection as proposed, the declines will not be limited to Thailand. In fact, an increase in the average age of dengue has been reported in several other countries in Southeast Asia that are experiencing reductions in birth and death rates [3]–[5]. Most countries in the region lag behind Thailand in the demographic transition [36]. The populations of Cambodia, Laos, Indonesia, and the Philippines are projected to age significantly in the coming decade [36]. Our work suggests than many of these countries will experience the same declines in the force of infection and shift in the age distribution of DHF cases that Thailand has experienced.

## Supporting Information

Alternative Language Abstract S1 Thai translation of the abstract by SI.
(0.08 MB PDF)

Text S1 Detailed methods and supplemental results.
(1.15 MB DOC)

## Abbreviations

CI: confidence interval
DF: dengue fever
DHF: dengue hemorrhagic fever
DSS: dengue shock syndrome

## References

[1] Kongsomboon K, Singhasivanon P, Kaewkungwal J, Nimmannitya S, Mammen MP (2004). Temporal trends of dengue fever/dengue hemorrhagic fever in Bangkok, Thailand from 1981 to 2000: an age-period-cohort analysis.. Southeast Asian J Trop Med Public Health.

[2] Halstead SB (2005). More dengue, more questions.. Emerg Infect Dis.

[3] Malavige GN, Velathanthiri VG, Wijewickrama ES, Fernando S, Jayaratne SD (2006). Patterns of disease among adults hospitalized with dengue infections.. QJM.

[4] Thai KT, Binh TQ, Giao PT, Phuong HL, Hung lQ (2005). Seroprevalence of dengue antibodies, annual incidence and risk factors among children in southern Vietnam.. Trop Med Int Health.

[5] Wichmann O, Hongsiriwon S, Bowonwatanuwong C, Chotivanich K, Sukthana Y (2004). Risk factors and clinical features associated with severe dengue infection in adults and children during the 2001 epidemic in Chonburi, Thailand.. Trop Med Int Health.

[6] Nagao Y, Koelle K (2008). Decreases in dengue transmission may act to increase the incidence of dengue hemorrhagic fever.. Proc Natl Acad Sci U S A.

[7] Anderson RM, May RM (1991). Infectious diseases of humans: dynamics and control..

[8] National Statistical Office T (1983). 1980 census..

[9] National Statistical Office T (2001). 2000 census..

[10] National Statistical Office T (2006). Statistical yearbook of Thailand, 2005..

[11] Williams JR, Manfredi P (2004). Ageing populations and childhood infections: the potential impact on epidemic patterns and morbidity.. Int J Epidemiol.

[12] Grenfell BT, Anderson RM (1985). The estimation of age-related rates of infection from case notifications and serological data.. J Hyg (Lond).

[13] Muench H (1959). Catalytic models in epidemiology..

[14] Earn DJD, Rohani P, Bolker BM, Grenfell BT (2000). A simple model for complex dynamical transitions in epidemics.. Science.

[15] Cummings DAT, Irizarry RA, Huang NE, Endy TP, Nisalak A (2004). Travelling waves in the occurrence of dengue haemorrhagic fever in Thailand.. Nature.

[16] Cummings DAT, Schwartz IB, Billings L, Shaw LB, Burke DS (2005). Dynamic effects of anti body-dependent enhancement on the fitness of viruses.. Proc Natl Acad Sci U S A.

[17] Ferguson N, Anderson R, Gupta S (1999). The effect of antibody-dependent enhancement on the transmission dynamics and persistence of multiple-strain pathogens.. Proc Natl Acad Sci U S A.

[18] Chareonsook O, Foy HM, Teeraratkul A, Silarug N (1999). Changing epidemiology of dengue hemorrhagic fever in Thailand.. Epidemiol Infect.

[19] National Statistical Office T (1991). 1990 census..

[20] National Oceanic and Atmospheric Administration (2004). National Oceanic and Atmospheric Administration Climate Anomaly Monitoring System..

[21] Farrington CP, Whitaker HJ (2003). Estimation of effective reproduction numbers for infectious diseases using serological survey data.. Biostatistics.

[22] Ferguson NM, Donnelly CA, Anderson RM (1999). Transmission dynamics and epidemiology of dengue: insights from age-stratified sero-prevalence surveys.. Philos Trans R Soc Lond B BiolSci.

[23] Burke DS, Nisalak A, Johnson DE, Scott RM (1988). A prospective study of dengue infections in Bangkok.. Am J Trop Med Hyg.

[24] Nisalak A, Endy TP, Nimmannitya S, Kalayanarooj S, Thisayakorn U (2003). Serotype-specific dengue virus circulation and dengue disease in Bangkok, Thailand from 1973 to 1999.. Am J Trop Med Hyg.

[25] Endy TP, Nisalak A, Chunsuttiwat S, Libraty DH, Green S (2002). Spatial and temporal circulation of dengue virus serotypes: A prospective study of primary school children in Kamphaeng Phet, Thailand.. Am J Epidemiol.

[26] Fischer DB, Halstead SB (1970). Observations related to pathogenesis of dengue hemorrhagic fever. Examination of age specific sequential infection rates using a mathematical model.. Yale J Biol Med.

[27] Nagao Y, Svasti P, Tawatsin A, Thavara U (2007). Geographical structure of dengue transmission and its determinants in Thailand. EpidemiolInfect.

[28] Torrence C, Compo GP (1998). A practical guide to wavelet analysis.. Bulletin of the American Meteorological Society.

[29] Wearing HJ, Rohani P (2006). Ecological and immunological determinants of dengue epidemics.. Proc Natl Acad Sci U S A.

[30] Schenzle D (1984). An age-structured model of pre- and post-vaccination measles transmission.. IMA J Math Appl Med Biol.

[31] Halstead SB (1994). Dengue in the health transition.. Gaoxiong Yi Xue Ke Xue Za Zhi.

[32] Reves R (1985). Declining fertility in England and Wales as a major cause of the 20th-century decline in mortality - the role of changing family-size and age structure in infectious-disease mortality in infancy.. Am J Epidemiol.

[33] Manfredi P, Williams JR (2004). Realistic population dynamics in epidemiological models: the impact of population decline on the dynamics of childhood infectious diseases. Measles in Italy as an example.. Math Biosci.

[34] Bureau of Epidemiology, Ministry of Public Health of Thailand (2000). Annual epidemiological surveillance report..

[35] Guzman MG, Kouri G, Bravo J, Valdes L, Vazquez S (2002). Effect of age on outcome of secondary dengue 2 infections.. Int J Infect Dis.

[36] United States Bureau of the Census (2008). International data base: population pyramids..

