# Supplementary material for: The Impact of the Demographic Transition on Dengue in Thailand: Insights from a Statistical Analysis and Mathematical Modeling
Source: PLoS Med. 2009 Sep 1;6(9):e1000139. doi: 10.1371/journal.pmed.1000139 (PMC2726436; doi:10.1371/journal.pmed.1000139)
Supplement: Alternative Language Abstract S1 — Thai translation of the abstract by SI. (0.08 MB PDF) [file pmed.1000139.s001.pdf]

The impact of the Demographic Transition on Dengue in Thailand

ผลกระทบของการเปลี่ยนผ่านโครงสร้างประชากรที่มีต่อโรคไข้เลือดออกเด็งกี่ในประเทศไทย

ความเป็นมา

จากรายงานโรคพบว่าอายุเฉลี่ยของผู้ป่วยโรคไข้เลือดออกในประเทศไทยเพิ่มขึ้น แม้ไม่ทราบสาเหตุที่แน่ชัดแต่คำอธิบายที่เป็นได้ คือ การลดลงของประชากรยุงนำโรค การที่คนถูกยุงกัดน้อยลง และผลจากการเปลี่ยนวิธีการรายงานผู้ป่วย ในการศึกษานี้ ผู้นิพนธ์นำเสนอสมมติฐานว่าการเปลี่ยนแปลงของโครงสร้างประชากรที่มีอัตราเกิดและอัตราตายที่ต่ำลงในประเทศไทย ทำให้การถ่ายทอดเชื้อไวรัสเด็งกี่ลดลงและส่งผลให้ระยะเวลาระหว่างการระบาดครั้งใหญ่ยาวนานขึ้น

วิธีการและผลการศึกษา

ทำการศึกษาข้อมูลเฝ้าระวังโรคจาก **72** จังหวัดในประเทศไทยเพื่อหาความสัมพันธ์ระหว่างความแรงของการติดเชื้อ (ดูอัตราเกิดโรค) กับโครงสร้างประชากรและตัวแปรอากาศ โดยทำการคำนวณความแรงของการติดเชื้อโดยใช้ข้อมูลการกระจายของอายุผู้ป่วยระหว่างปีพ.ศ.**2528-2548** ผลการศึกษาพบว่าความแรงของการติดเชื้อลดลงร้อยละ **2** ต่อปีตั้งแต่ช่วงประมาณปี **2522-2523** ที่มีอัตราป่วยสูงสุด ได้ผลตรงกันข้ามกับการศึกษาอื่นเร็วๆนี้ ที่พบว่าอัตราอุบัติการณ์ไข้เลือดออกเด็งกี่เพิ่มขึ้นในประเทศไทย ในการศึกษานี้พบว่าอัตราอุบัติการณ์ลดลงเล็กน้อยอย่างมีนัยสำคัญทางสถิติในจังหวัดส่วนใหญ่ตั้งแต่ปีพ.ศ.**2528** เป็นต้นมา โดยปัจจัยที่มีผลต่อการเปลี่ยนแปลงความแรงของการติดเชื้อและค่าเฉลี่ยของความแรงของการติดเชื้อ ได้แก่ มัธยฐานอายุของประชากร ซึ่งจากการใช้แบบจำลองทางคณิตศาสตร์สาธิตการถ่ายทอดเชื้อของไวรัสเด็งกี่ แสดงให้เห็นว่าการลดของอัตราเกิดและการเปลี่ยนแปลงโครงสร้างอายุประชากร สามารถอธิบายการเพิ่มขึ้นของอายุผู้ป่วย รวมทั้งการลดลงของความแรงของการติดเชื้อ และการเพิ่มระยะห่างของลูกคลื่นอัตราอุบัติการณ์เกิดโรคในช่วงหลายๆปี เมื่อทำการควบคุมตัวแปรอื่นๆ

สรุปผลการศึกษา

อัตราเกิดและอัตราตายที่ต่ำลงส่งผลทำให้การไหลเข้าของประชากรผู้มีความไวรับลดลง และทำให้เพิ่มช่วงเวลาของการมีภูมิคุ้มกันในประชากร การเพิ่มขึ้นของสัดส่วนประชากรที่มีภูมิคุ้มกันต่อโรคทำให้เพิ่มโอกาสที่ยุงจะดูดเลือดจากประชากรกลุ่มนี้ และส่งผลทำให้ความแรงของการติดเชื้อลดลง ผู้ศึกษาพบว่าสัดส่วนวิกฤติของการรับวัคซีนลดลงเล็กน้อยจากร้อยละ **85** เป็น **80** แม้ว่าความแรงของการถ่ายทอดเชื้อจะลดลงครึ่งหนึ่งก็ตาม ในคู่มือแนวทางดูแลผู้ป่วยทางคลินิกควรต้องเพิ่มเรื่องการดูแลผู้ป่วยไข้เลือดออกในคนที่มีอายุมากขึ้น ประเทศอื่นๆในภูมิภาคที่ยังตามหลังประเทศไทยในเรื่องโครงสร้างประชากร คงจะพบการเพิ่มขึ้นของอายุประชากรเช่นเดียวกับประเทศไทยในอนาคต สำหรับสมมติฐานเรื่องผลกระทบของโครงสร้างประชากรต่อความแรงของการถ่ายทอดเชื้อมีการศึกษาในโรคอื่นๆมาก่อน แต่เท่าที่ผู้นิพนธ์ทราบ การศึกษานี้สามารถเสนอให้เห็นปรากฏการณ์นี้ในโรคไข้เลือดออกได้เป็นครั้งแรก
