# Supplementary material for: The Impact of the Demographic Transition on Dengue in Thailand: Insights from a Statistical Analysis and Mathematical Modeling
Source: PLoS Med. 2009 Sep 1;6(9):e1000139. doi: 10.1371/journal.pmed.1000139 (PMC2726436; doi:10.1371/journal.pmed.1000139)
Supplement: Text S1 — Detailed methods and supplemental results. (1.15 MB DOC) [file pmed.1000139.s002.doc]

**Supporting Information**

Age Standardization

We used direct age adjustment to estimate mean, median and mode age of dengue cases adjusted for differences in age structures in Thailand over time. We applied age specific incidence rates calculated in each year to our standard population, Thailand in 2000.

Force of Infection

Methods for estimating the force of infection, *λ*, for infections from age stratified serological or case data for single serotype pathogens are well established and have been reviewed elsewhere (1, 2). Estimation of *λ* for dengue is complicated by the unknown role of pre-existing heterotypic immunity in promoting infection or transmission among individuals experiencing their second infection (3). The use of age stratified case reports to estimate the force of infection is further complicated by a lack of clear knowledge of the age specific risk of severe disease upon infection. Ferguson et al., developed methods for estimating the force of infection from age stratified serological data describing the rates of primary and multi-typic serological responses to dengue that incorporated multiple assumptions of the role of pre-existing immunity (3). We have adapted these methods in order to estimate forces of infection from age stratified incidence data. We assume that the age distribution of incident disease is representative of the age distribution of secondary infections. We also assume that everyone in the population is infected twice in their lifetime. This assumption is supported by age stratified serological surveys that find that nearly 100% of individuals aged 20 and above show evidence of immunity to multiple serotypes of dengue (4). We assume, then, that the cumulative proportion of age specific incidence as a function of age provides an estimate of the proportion of each age group that has experienced two infections of dengue (which we denote as a function of time, *z**(a,t)).*

The fraction of the population susceptible to all dengue serotypes at age *a* and *t*, *x(a,t)* is given by

$x(a,t)=e^{-\int_{0}^{a} \sum_{i} \lambda_{i}(a-\tau,t-\tau)d\tau}$ (1)

where *λ_i_* is the force of infection of serotype *i*. The proportion of individuals of age a who have been infected with only serotype *i* at time t, but still susceptible to all other serotypes is denoted *z_i_(a,t)* and is given by:

$z_{i}(a,t)=x(a,t)\left[e^{\int_{0}^{a} \lambda_{i}(a-\tau,t-\tau)d\tau}-1\right]$ (2)

Also

(3)

We denote multi-typic infections *z**(a,t)* and note that *z**(a,t)=1-x(a,t)- Σzi(a,t)*.

We denote the cumulative proportion of age specific incidence at time t and age a *n**(a,t).* We assume that this gives an estimate of those people who have experiences two infections at age a and time t. We denote the proportion of age a at time t who are completely susceptible to dengue, *n_00_(a,t)*, those that have been infected with just one serotype, *i*, *n_i0_(a,t)*. Age specific incidence provides an eprovide estimates of *x(a,t)*, *z_i_(a,t)* and *Σz**(a,t)* respectively. Maximum likelihood estimates of *λ_i_* can be found by maximizing equation (4) where *n**(a_i_,t)* is given by the cumulative proportion of cases occurring up to age *a_i_* for year *t.* Only the sum of *n_00_(a_i_,t)* + *Σn_i0_(a_i_,t)* is given by the data by *1-n**(a_i_,t)*. Given our assumptions, the log likelihood of cumulative proportion of age specific incidence as a function of the parameters *λ_i_* is given by:

(4)

We calculate the maximum likelihood estimates of *λ_i_(t)* for 40 years, each year for which we have data (1985-2005) as well as years for which the age cohorts represented in the data are exposed (1965-1984), making the simplifying assumption that each person in each province has been at risk of infection in that province their entire life. We assume that *λ_i_(t)= λ(t)* for all *i*.

In addition to a model with unique *λ* for each year in which individuals in the age cohorts considered have exposure, we also use a model for which *λ* varies by year and age. We also assume that the force of infection encountered by individuals of age group *a* at time *t*, *λ(a,t)* is given by *κ(a)+ λ(t)* where *κ(a)* is an age specific additive factor (model 3) or *κ(a)*λ(t) where κ(a)* is an age specific multiplier (model 4) that is constant through time and shared between all provinces.

Model 1

1 parameter

Model 2

41 parameters for each province, forces of infection for each of the years 1965-2005

Model 3

40 parameters for each province

10 age specific parameters shared across all provinces

Model 4

40 parameters for each province

10 age specific parameters shared across all provinces

We used a standard numerical optimization tool to find maximum likelihood parameters of each model (a quasi-Newton method (L-BFGS-B) implemented in the R statistical package) [38].

We used the likelihood ratio test to determine if additional parameters statistically significantly improved the fit of the model. We calculated the ratio of the likelihood of each model. We calculated the test statistic *2(LL_larger_-LL_reduced_), where LL_larger_* is the log likelihood of the model with more parameters and LL_reduced_ is the maximum log likelihood of the model with fewer parameters. We assume this statistic is *χ^2^* distributed with degrees of freedom equal to the difference in the number of parameters between the two models. Table S1 shows the results of likelihood ratio tests comparing the models. Confidence intervals appearing in Figure 2 of the text were calculated by calculating profile likelihoods of each model parameter. Figure S1 shows the age specific multipliers estimated using model 4.

Estimates of the force of infection are highly correlated. Figure S2 shows plots of one model’s estimates versus other models. Linear regression of one model’s estimates of the mean of the force of infection on another’s yields: R^2^ of 0.96 for Model 3 on Model 2, R^2^ of 0.92 for Model 4 on Model 2, R^2^ of 0.96 for Model 4 on Model 3. Linear regression of one models estimates of the change in mean of the force of infection on another’s yields: R^2^ of 0.62 for Model 3 on Model 2, R^2^ of 0.66 for Model 4 on Model 2 and R^2^ of 0.93 for Model 4 on Model 3.

Figure S3 shows the fit of a model that includes a factor that modifies the force of infection of individuals who have already experienced one infection. We estimated these individuals to have 1.43 (95% CI 1.3, 1.62) times the risk of those who have experienced no infections. The model also includes age specific multiplicative factors. The estimates of multiplicative factors do not change significantly with the inclusion of this factor nor do the time specific forces of infection.

Comparison of Estimates of the Force of Infection in Rayong from age specific serological data and age specific incidence data

A serological survey conducted in Rayong province in 1980 provides an opportunity to compare estimates of the force of infection derived from serological data and estimates derived from age specific incidence data [Sangkawibha N, 1984]. Figure S4 presents estimates from an analysis by Ferguson et al (filled circles) and model 4 using age data from 1980-2005 (open circles) [Ferguson, 1999]. The sum of the force of infection across all serotypes is shown for each year 1969-1979. The time range shown is dictated by the estimates available in Ferguson et al [Ferguson, 1999]. The mean of the sum of the force of infection across all serotypes for the entire range in Ferguson’s analysis is 0.36. The mean of the sum of the force of infection across all serotypes derived from model 4 is 0.49 (95% CI 0.29, 0.60). The point estimate from the Ferguson paper overlaps with the confidence interval estimated using model 4.

Wavelet Transform

Continuous wavelet transforms were performed using the Morlet wavelet. A complete description of methods can be found elsewhere [26,39]. We estimated the period of multiannual oscillations by calculated the weighted average of periods between 18 and 60 months using wavelet coefficients as weights.

Regressions

Weighted linear regression was used to test for associations between the force of infection and socio-demographic and climate covariates. Climate covariates were included to assess any potential confounding with socio-demographic covariates. Regression models were weighted by the inverse of the variance of the estimated dependent variable (e.g. linear trend in force of infection over time, or linear trend in multi-annual periodicity). Model 4 was used for the results presented in the main body of the paper.

The results of regression with forces of infection estimated using model 2 are shown in Table S2 and S3. Results of regression with forces of infection estimated using model 3 are shown in Table S4 and S5.

Simulations

Three non-age specific deterministic models were used to examine the impact of changes in birth and death rates on the period of oscillations. These models were of the form,

$$\begin{matrix} \frac{dx}{dt}=\sum_{i=1} \beta x\left(y_{i}+\varphi\sum_{j\neq i} y_{ji}\right)+\left(1-x\right)\mu\\ \frac{dy_{i}}{dt}=\beta x\left(y_{i}+\varphi\sum_{j\neq i} y_{ji}\right)-\left(\sigma+\mu\right)y_{i} \\ \frac{dc_{i}}{dt}=\sigma y_{i}-\left(\delta+\mu\right)c_{i} \\ \frac{dz_{i}}{dt}=\delta c_{i}-\sum_{j=1,j\neq i} \beta z_{i}\left(y_{j}+\varphi\sum_{k\neq j} y_{kj}\right)-\mu z_{i} \\ \frac{dy_{ij}}{dt}=\beta z_{i}\left(y_{j}+\varphi\sum_{k\neq j} y_{kj}\right)-\left(\sigma+\mu\right)y_{ij} \\ \frac{dz_{**}}{dt}=\sigma y_{ij}-\mu z_{**} \end{matrix}$$

where *x* represents the fraction of the population susceptible to all serotypes, *y­_i_* those currently infectious with serotype *i* having never been infected with other serotypes, *c_i_* those that have been infected with serotype *i*, recovered and are temporarily immune to other serotypes and *z_i_* infected with serotype *i* who have recovered and are immune to serotype *i* but susceptible other serotypes. *y_ij_* represents those currently infectious with serotype *j* having been infected with serotype *i*. Finally, *z*** represents those that have been infected with two serotypes and gained broadly cross-reactive immunity to all serotypes. The transmission coefficient, β, indicates the rate at which infectious contacts are made. Values between 200 and 600 were used. The enhancement factor Φ indicates the factor by which secondary infections are more infectious than primary infectious. The length of the infectious period is indicated by 1/σ. The average life span is indicated by 1/μ. The length of the short-term cross-protected period is 1/δ. Simulations included four serotypes. Separate models were considered incorporating 1) seasonality of transmission, 2) antibody dependent enhancement and 3) short-term cross immunity. Seasonality of transmission was modeled using a sin function with peak transmission coefficients 4% higher than mean levels. Age specific models used the same model structure but divided the population into 9 two year age classes and one age class representing those 18 years of age and older.

Age Specific Simulations incorporating age dependent and age independent mortality

We investigated the impact of changing demography on the magnitude of total incidence across the entire population using two age specific models, model A1, the model used to produce figure 7 in the main body of the manuscript which uses age independent mortality rates and Model A2, which uses age dependent mortality rates. In model A2, the first 9 age classes mortality rates are 0. All mortality occurs among individuals in the oldest age class. The prevalence of secondary infection in model A1 and Model A2 is shown in Figure S5 for a model using Beta=350 and varying birth and death rates as shown. Enhancement is not included in this model nor is seasonality.

Model A1

Simulation of two serotype model with age independent mortality

Model A2

Simulation of two serotype model with age dependent mortality

For age classes 1-9: (note mortality rate=0)

For age class 10:

**Table S1**: **Models used to estimate the force of infection from the age distribution of dengue cases**

The type of model, number of parameters, and performance of each model fit to the age specific incidence data are shown. Performance is measured as -2 times the difference between the log likelihood of a model using just one force of infection for all years and the model indicated. P-values for tests for a significant difference between each model and more complicated models are shown in the last three columns.

| 1 | Parameters | Outcome of Likelihood Ratio Test comparing outcome to Model 1  (p value) | LR to Model 2 | LR to Model 3 |
| --- | --- | --- | --- | --- |
| Constant Force of Infection | 1 | 1 (NA) |  |  |
| Time specific forces of infection | 41 parameters, *λ(t)* for each year 1965-2005 | p<<0.001 | 1 |  |
| Time specific forces of infection and additive age specific factors | 51 parameters, 41 *λ(t)* for each year 1965-2005 and 10 age specific factors | p<<0.001 | p<<0.001 | 1 |
| Time specific forces of infection and additive age specific factors | 51 parameters, 41 *λ(t)* for each year 1965-2005 and 10 age specific factors | p<<0.001 | p<<0.001 | p<<0.03 |

**Table S2**: Results of univariate linear regression of Force of Infection estimated using Model 2 on demographic, socioeconomic and climatic characteristics of provinces

| Independent Variable | Regression Coefficients  (95% Confidence Interval) |
| --- | --- |
| Median age | -1.3x10^-3^ (-2.0x10^-3^, -5.4x10^-4^) |
| Mean Household size | 7.4x10^-3^ (1.9x10^-3^, 1.3x10^-2^) |
| Birth Rate (per 1000) | 5.7x10^-4^ (1.5x10^-4^, 9.9x10^-4^) |
| Proportion of population under 15 years of age | 0.05 (0.02, 0.09) |
| Gross Provincial Product (Baht per capita) | 7.9x10^-8^ (2.6x10^-8^, 1.3x10^-7^) |
| Percent of homes with sanitation | -1.8x10^-4^ (-3.1x10^-4^, -4.0x10^-5^) |
| Percent of provincial population in urban areas | 1.1x10^-4^ (6.8x10^-6^, 2.1x10^-4^) |
| Average Rainfall (mm/month) | 5.0x10^-5^ (3.9x10^-6^, 9.5x10^-5^) |
| Latitude (Degrees) | -1.5x10^-3^ (-1.9x10^-3^,-1.1x10^-3^) |

School attendance, percent of homes constructed with permanent materials and average temperature were not statistically significantly associated with the mean force of infection

**Table S3**: Results of univariate linear regression of change in the force of infection estimated using Model 2 from 1985-2005 on demographic, socioeconomic and climatic characteristics of provinces

| Independent Variable | Regression Coefficients  (95% Confidence Interval) |
| --- | --- |
| Change in Median age | -0.06 (-0.09, -0.02) |
| Change in percentage of homes constructed with permanent materials | -0.02 (-0.03, -0.005) |

Change in school attendance, household size, percent of provincial population in urban areas, birth rate, gross provincial product, average rainfall and average temperature were not statistically significantly associated with the change in the mean force of infection

**Table S4**: Results of univariate linear regression of Force of Infection estimated using Model 3 on demographic, socioeconomic and climatic characteristics of provinces

| Independent Variable | Regression Coefficients  (95% Confidence Interval) |
| --- | --- |
| Median age | -1.3e-3 (-1.9e-3, -6.9e-4) |
| Mean Household size | 7.7e-3 (3.0e-3, 1.2e-2) |
| Birth Rate (per 1000) | 5.3e4 (1.7e4, 9.0e4) |
| Proportion of population under 15 years of age | 5.8e-2 (3.0e-2, 8.8e-2) |
| Gross Provincial Product (Baht per capita) | 5.1e-8 (4.3e-10, 1.0e-7) |
| Percent of homes with sanitation | -1.9e-4 (-3.0e-4, -7.7e-5) |
| Average Rainfall (mm/month) | 4.6e-5 (1.1e-5, 8.1e-5) |
| Latitude (Degrees) | -1.2e-3 (-1.6e-3, -8.9e-4) |

School attendance, percent of provincial population in urban areas, percent of homes constructed with permanent materials and average temperature were not statistically significantly associated with the mean force of infection

Covariates significant in multiple linear regression: Median age -2.2e-3 (95% CI

-4.7e-3, -3.2e-5), Latitude -1.3e-3 (-2.4e-3, -2.4e-4), Birth Rate -9.1e-4 (95% CI -1.8e-3, -5.0e-6)

**Table S5**: Results of univariate linear regression of change in the force of infection from 1985-2005 estimated using Model C on demographic, socioeconomic and climatic characteristics of provinces

| Independent Variable | Regression Coefficients  (95% Confidence Interval) |
| --- | --- |
| Change in Median age | -0.04 (-7.0e-2, -8.3e-3) |
| Change in percentage of homes constructed with permanent materials | -0.01 (-2.7E-2, -5.3e-3) |

Change in school attendance, household size, percent of provincial population in urban areas, birth rate, gross provincial product, average rainfall and average temperature were not statistically significantly associated with the change in the mean force of infection

Covariates significant in multiple linear regression: Median age (-0.05 (95% CI, -9.9e-2, -5.7e-3 percent of homes constructed with permanent materials (-0.01 (95% CI -0.02, -5.4e-4)

**Figure S1**

Age specific multipliers estimated using Model 4.

**
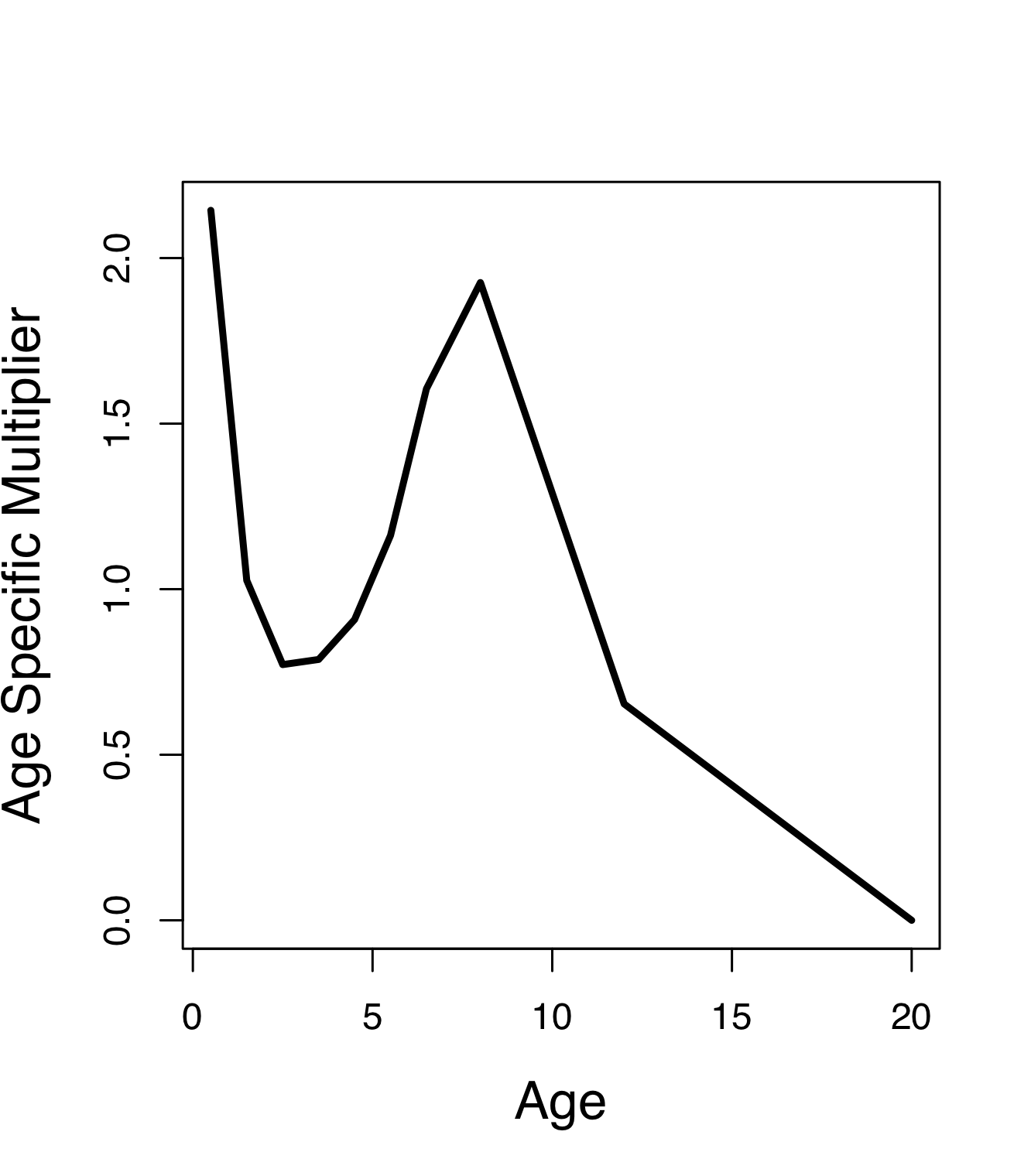
**

**Figure S2**

Comparison of mean λ(t) and the change in λ(t) over time estimated using three different models. **A.** Mean of λ(t) for the period 1985 to 2005 estimated using model 3 versus those estimated using model 2. **B.** Mean of λ(t) from model 3 versus model 2. **C.** Mean of λ(t) from model 4 versus model 3. **D.** Change in λ(t) from 1985 to 2005 estimated using model 3 versus model 2. **E.** Change in λ(t) from model 4 versus model 2. **F.** Change in λ(t) from model 4 versus model 3.


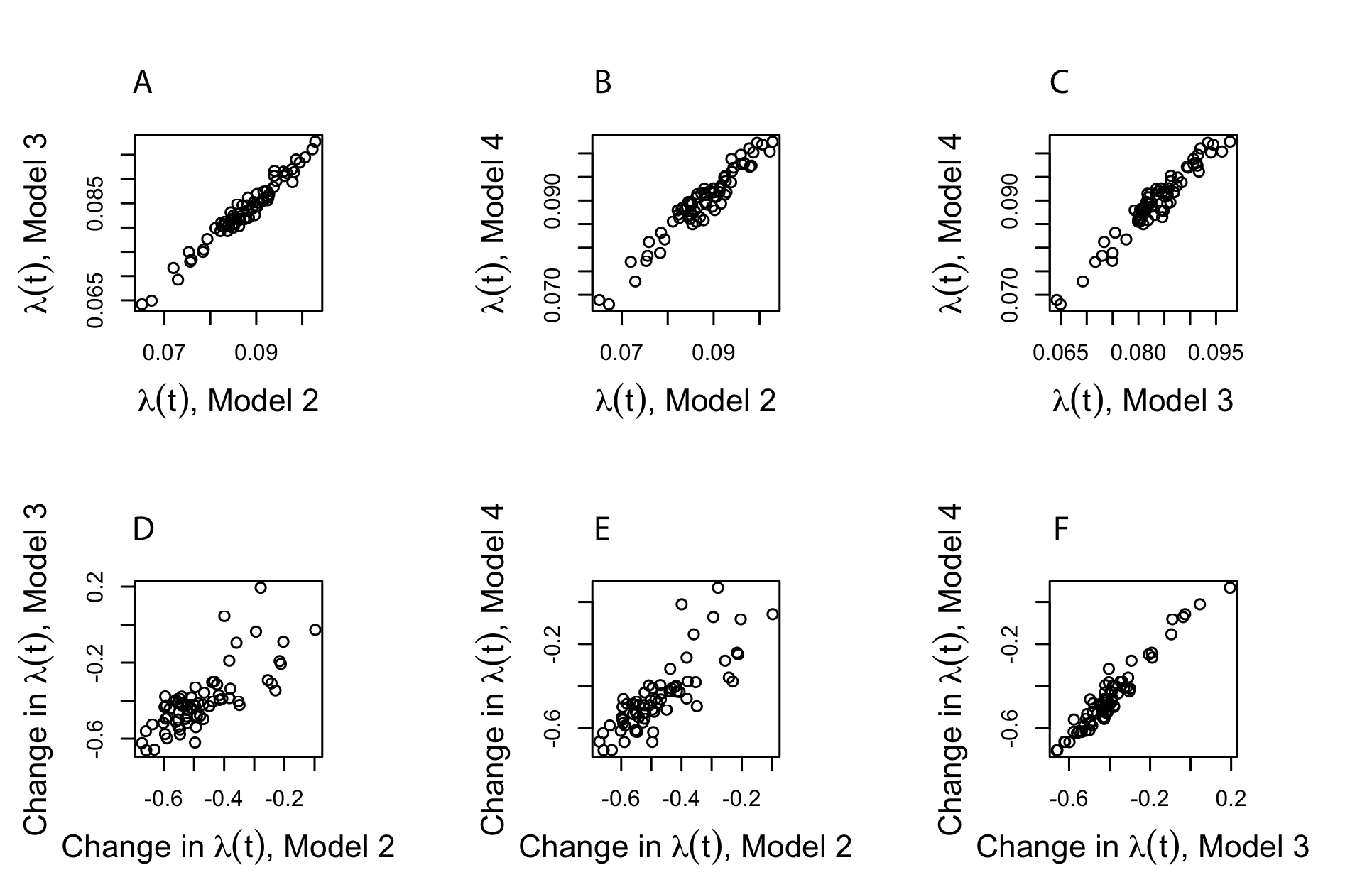


**Figure S3**

Fit of model including age specific multiplicative factors and a factor modifying the hazard of individuals having already experienced one infection to data 1985, 1990, 1995, 2000, 2005, Model 2, 3 and 4

**
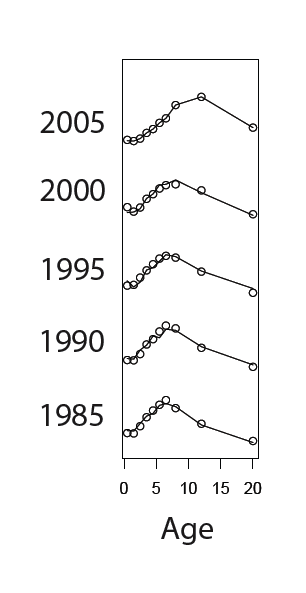
**

**Figure S4**

Estimated force of infection using serological data from (4) and our age specific incidence data for Rayong province. Mean forces of infection over the entire interval are not statistically significantly different.


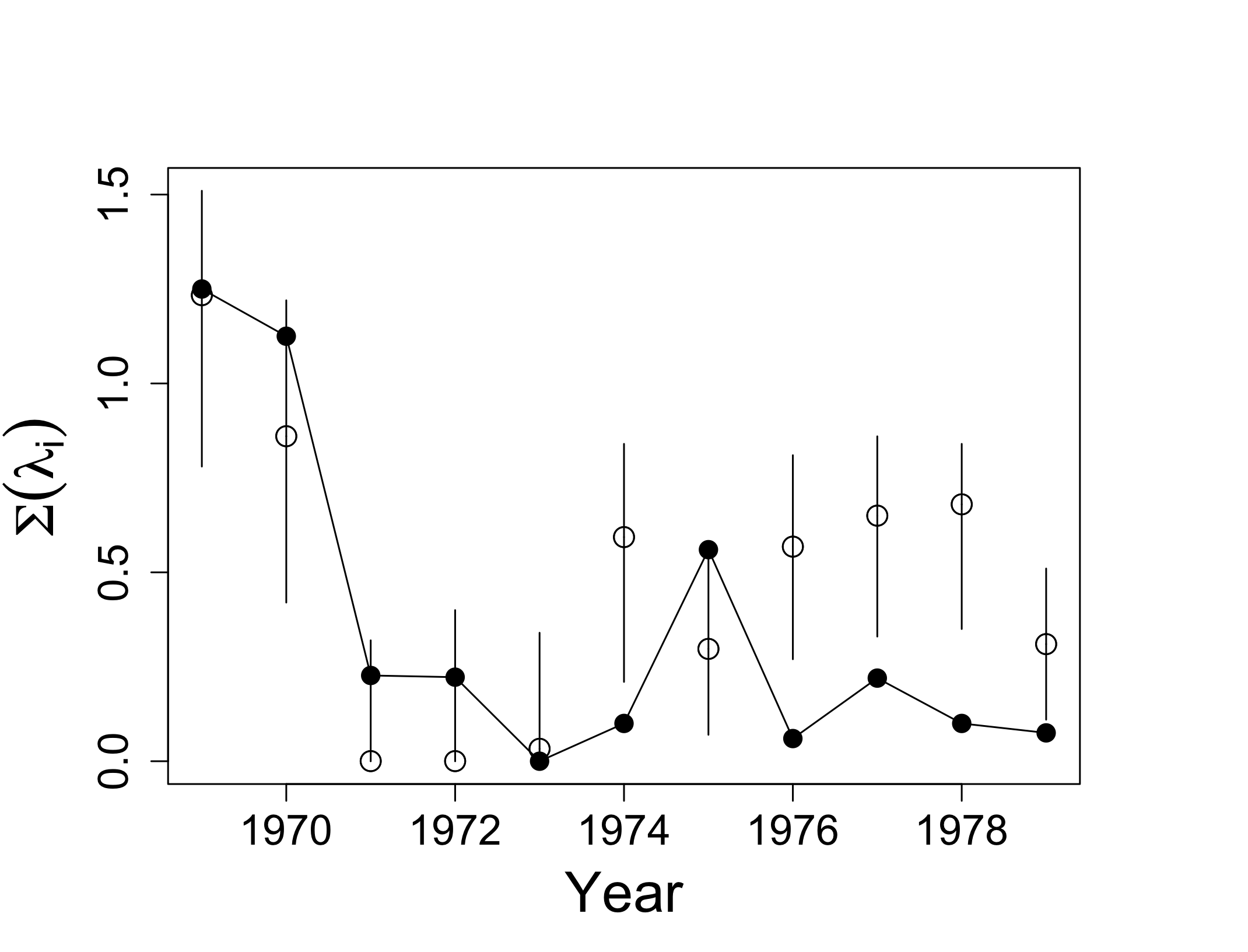


**Figure S5**

Prevalence of secondary infection under different rates of birth and death (x-axis). Shown our results using Model A1 (black curve) and Model A2 (blue curve).


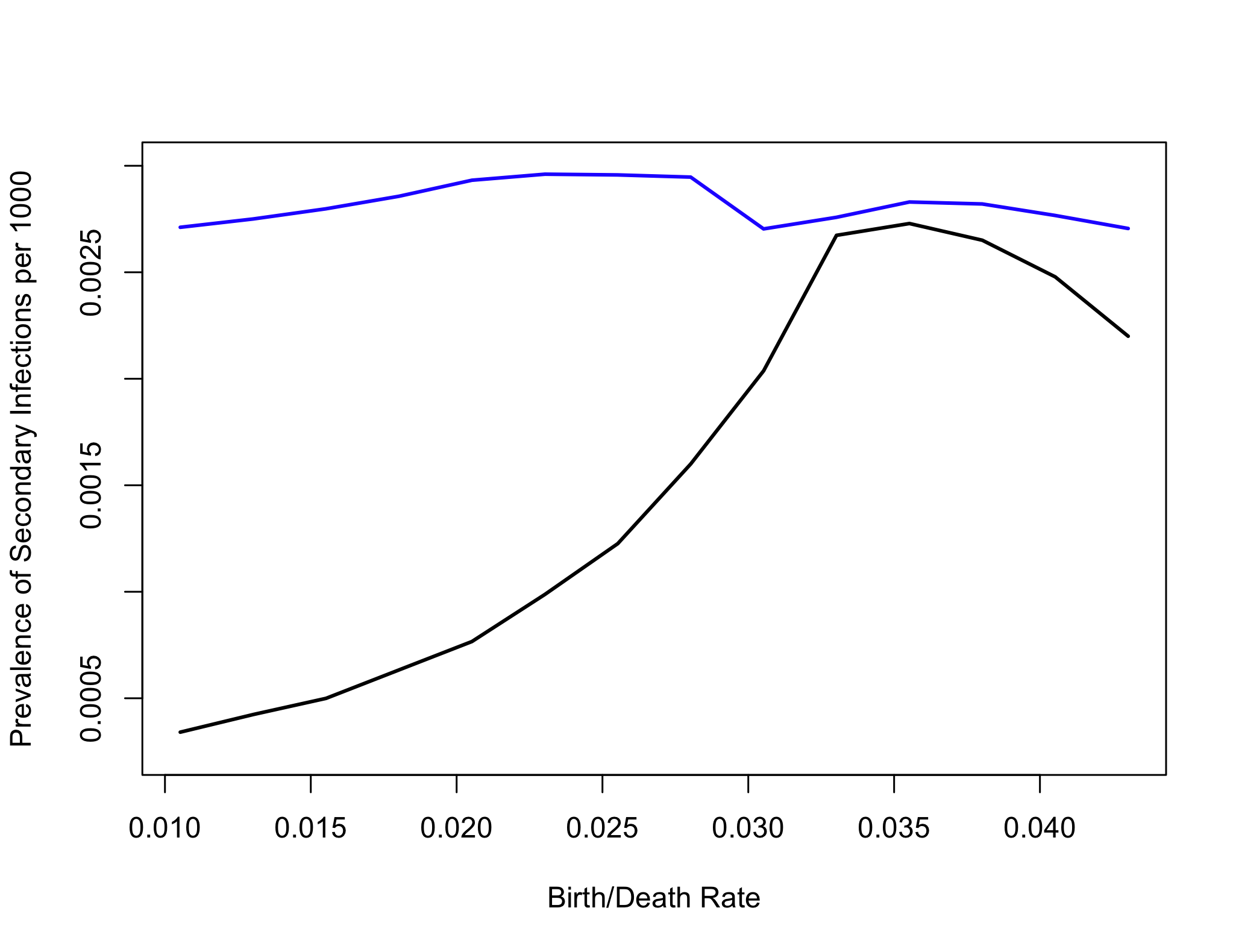


References

1. Grenfell BT, Anderson RM. The Estimation of Age-Related Rates of Infection from Case Notifications and Serological Data. Journal of Hygiene 1985;95:419-36.

2. Heisey DM, Joly DO, Messier F. The fitting of general force-of-infection models to wildlife disease prevalence data. Ecology 2006;87:2356-65.

3. Ferguson NM, Donnelly CA, Anderson RM. Transmission dynamics and epidemiology of dengue: insights from age-stratified sero-prevalence surveys. PhilosTransRSocLond B BiolSci 1999;354:757-68.

4. Sangkawibha N, Rojanasuphot S, Ahandrik S, et al. Risk-Factors in Dengue Shock Syndrome - A Prospective Epidemiologic-Study in Rayong, Thailand .1. the 1980 Outbreak. American Journal of Epidemiology 1984;120:653-69.
